# Screening of Hepatitis G and Epstein-Barr Viruses Among Voluntary non Remunerated Blood Donors (VNRBD) in Burkina Faso, West Africa

**DOI:** 10.4084/MJHID.2013.053

**Published:** 2013-09-02

**Authors:** Issoufou Tao, Cyrille Bisseye, Bolni Marius Nagalo, Mahamoudou Sanou, Alice Kiba, Guzin Surat, Tegwindé Rebeca Compaoré, Lassina Traoré, Jean Baptiste Nikiema, Virginio Pietra, Jean-Didier Zongo, Jacques Simpore

**Affiliations:** 1 Pietro Annigoni Biomolecular Research Centre (CERBA)/LABIOGENE, University of Ouagadougou, Burkina Faso.; 2 National Blood Transfusion Centre, Ouagadougou, Burkina Faso.; 3 Laboratory of genetics, University of Ouagadougou, Burkina Faso.; 4 Department of Biology, University of Sciences and Techniques of Masuku (Franceville), Gabon

## Abstract

In most sub-Saharan countries screening of blood-transmitted infections includes mainly HIV, HBV, HCV and syphilis. Many viruses such as Hepatitis G (HGV) and Epstein-Barr virus (EBV) which also carry a risk of transmission by blood transfusion raise the question of the extent of screening for these pathogens. This work aims to evaluate the prevalence of HGV and EBV in first-time blood donors in Ouagadougou.

The prevalence of HGV and EBV in 551 blood donors was 7.4% and 5.4% respectively. HGV prevalence was significantly higher in blood donors with hepatitis B antigens and positive for HCV compared to donors negative for HCV and no hepatitis B antigens (respectively p<0.001 and p=0.004). EBV prevalence was higher among blood donors of < 20 years age group. HBV and HCV positive individuals are not eligible for blood donation.

This study shows significant results with regard to the prevalence of HGV and EBV prevalence in blood donors in Burkina Faso and emphasizes the need for a general screening.

## Introduction

Despite the remarkable progress in detecting transfusion-transmissible infectious pathogens, blood transfusion still remains a significant mode of transmission of infectious agents. Given the wide variety of blood-borne agents, there is an urgent need to determine their epidemiology.

Burkina Faso suffers from a shortage of blood products, with merely 3.1 units per 1,000 habitants as opposed to 10 units per 1,000 inhabitants required,^1^ due to malaria, malnutrition, emergency surgery, childbirth and traffic accidents.^2^–^4^

The main infectious diseases detected among blood donors are human immunodeficiency virus (HIV), along with Hepatitis B and C viruses (HBV and HCV). The screening of viruses such as Hepatitis G (HGV), Epstein - Barr virus (EBV), Cytomegalovirus (CMV) and Human Lymphotropic T Cell Virus (HLTV) remains unaddressed.

In a recent report GBV-C (HGV), GBV-A and GBV-D have been classified in a new genus Pegivirus within the Family Flaviviridae.^5^ The clinical role of HGV is not entirely clarified yet. HGV is not associated with any hepatic morphological modifications.^6^ However, a recent study showed an association between HGV infection with aplastic anemia and extrahepatic diseases.^7^

The prevalence of HGV in the world varies from 2% in the United States of America to 12.2% in Brazil and 18% in Egypt and South Africa, respectively.^8^ The virus is mainly transmitted via infectious blood and parenterally,^9^,^10^ while sexual transmission lacks significant evidence.^8^

Another neglected virus not diagnosed among blood donors in Burkina Faso is EBV. In immunocompetent individuals EBV is in a latent phase; and infected people show no clinical symptoms. Indeed, 90% of adults are EBV asymptomatic carriers. In immunosuppressed individuals the virus is reactivated and becomes oncogenic.^11^ EBV is etiologically associated with Burkitt’s lymphoma (a B cell-derived tumor), nasopharyngeal carcinoma,^12^ Hodgkin’s lymphoma^13^ and breast cancer.^14^–^16^ However, its mode of transmission remains controversial.

In Africa, early infections with EBV have been described in children, while in developed countries the primary infection is delayed until adulthood.^17^

In Burkina Faso, the prevalence of HGV and EBV are unknown to date. This study aims to determine the prevalence of HGV and EBV among blood donors in Burkina Faso and examine sociodemographic factors associated with both viral infections.

## Materials and Methods

### Blood donors

Blood samples were collected using standard procedures from voluntary non remunerated blood donors (VNRBD) at the Regional Blood Transfusion Centre of Ouagadougou (RBTC) between February and September 2012. VNRBD were all healthy subjects, selected after responding to a panel of questions comprising a medical history. Healthy individuals aged 17–65 years and over 50 kg were eligible for blood donation.

All samples were screened for hepatitis B virus surface antigen (HBsAg), antibodies to HIV, Treponema pallidum and HCV. Samples were kept at −20 °C for further analysis.

This study was approved by the CERBA/Saint Camille Ethics Committee. The consent of all study subjects was obtained before blood collection.

### Serological analysis

HBsAg and antibodies to HCV and HIV were detected using a fourth generation ELISA (ARCHITECT-i1000SR-ABBOTT, Santa Clara, California, United States of America).

All reactive samples for HBsAg, HCV and HIV were re-tested using a second ELISA (Bio-Rad, Marnes la Coquette, France). A result was considered positive if both the first and second tests were positive. Antibodies to Treponema pallidum were detected using a rapid plasma reagin (RPR) test (Cypress Diagnostics, Langdorp, Belgium) and confirmed with a Treponema pallidum haemagglutination (TPHA) test (Cypress Diagnostics).

### Hepatitis G virus RNA extraction and reverse transcription

Viral RNA was extracted from 140 μL of plasma using the QIAmp viral RNA extraction kit (Qiagen, Hilden, Germany) following the manufacturer’s instructions and was reverse transcribed using the HGV 340/625 IC PCR kit (Reverse transcription-PCR) with electrophoretic Detection (Sacace Biotechnologies, Como, Italy).

### EBV DNA extraction and amplification

Viral DNA was extracted from 200 μL of plasma using the QIAmp viral DNA extraction kit (Qiagen, Hilden, Germany) following the manufacturer’s instructions. The extracted DNA was used for PCR amplification using the EBV-290 PCR kit with electrophoretic Detection (Sacace Biotechnologies, Como, Italy).

### Statistical analysis

Data were analyzed using EPI-Info version 6.04 dfr (CDC, Atlanta, United States of America). A chi-square test was applied to compare proportions. Odds ratio was calculated to determine risk factors associated with HGV. P-values <0.05 were considered statistically significant.

## Results

We recruited 551 blood donors of whom 84.2% were men and 15.8% were women and the majority (61.9%) was between 20 and 29 of age. Five hundred forty eight subjects were first-time blood donors (99.5%), while 3 individuals were repeat donors (0.5%). Most of the blood donors belonged to blood group O (43.9%) and 92.7% were rhesus positive. The subjects were mainly recruited from urban settings (77.1%) Table 1. The overall seroprevalence of HBV, HCV, HIV and Treponema pallidum were of 12.9%, 3.3%, 2.2% and 2.7%, respectively (Table 2).

Screening for HGV in blood donors by viral RNA showed that 41 (7.4%) were positive. The prevalence of HGV infection was of 7.3% (40/548) in first time donors. HGV was principally associated with HBV (5.6%), HCV (1.0%) and syphilis (0.4%)

No co-infection of HGV and HIV was observed among the blood donors (Table 2).

As shown in Table 3, the prevalence of HGV was not related to sex (7.5 vs 6.9%) and age group. This prevalence was similar in urban compared to rural areas; but was five and seven times higher in HBV and HCV-positive blood donors compared to HBV and HCV-negative blood donors (p < 0.001; p=0.004).

EBV viral DNA was found in 5.4% (30/551) of blood donors. No significant statistical association was observed between EBV and the socio-demographic characteristics. EBV prevalence was studied in HIV and syphilis positive subjects as well as negative subjects. EBV prevalence was similar in HIV seropositive (8.3%) compared to HIV seronegative (6.1%) individuals, while it was higher among blood donors of < 20 years old age group compared with 20–29 age groups (p<0.001) (Table 3).

## Discussion

To this date, HGV prevalence is still unknown in Burkina Faso in blood donors as well as in the general population. Previous studies showed that the worldwide prevalence of HGV among blood donors is between 0.9 and 10%.^18^,^19^

This study documents that 7.4% of the blood donors are HGV carriers. This high prevalence of HGV is in accordance with previous studies which reported that HGV was 10 times higher in Africa than anywhere else.^8^ Different HGV prevalences have been reported in Africa and elsewhere. In healthy Kuwaitian, Jordanian and Tunisian blood donors the HGV prevalence of 24.6; 9.8% and 5.3%, have been reported, respectively.^20^,^21^ In central Africa, HGV prevalence of 10.3% and 12.6% was also found among pregnant women.^22^,^23^

The presence of HGV viral RNA was significantly higher in blood donors who had hepatitis B surface antigens (HBsAg) and were positive to anti-HCV compared to blood donors who were negative for HBsAg and anti-HCV. In fact, it is shown that HCV and HGV risk factors are very similar.^24^ A study showed that the frequency of anti-HCV antibodies was higher among HGV positive compared with HGV negative subjects.^25^ HBsAg and anti-HCV positive individuals are not eligible for blood donation. In this study, infection by HGV was not associated with age, either with the place of donation or sex. The high prevalence of HGV in blood donors who are HBsAg positive in Burkina Faso confirms that the infection is higher in patients at risk such as dialysis patients.^26^–^28^

We report for the first time the prevalence of EBV in blood donors in Burkina Faso, The prevalence of 5.4% found in a our study is less than the prevalence of 20% reported by Adjei et al^13^ in blood donors in Ghana. This difference could be due to the type of diagnostic used in these two studies: viral DNA screening and anti-EBV serology. A correlation between EBV and HIV-1 infection was also found in the same study. Here, we report a lack of correlation between EBV and HIV-1 infection. This could be explained by the low number of HIV-1 positive subjects studied.

EBV prevalence was higher in the age group <20 years. This is in accordance with previous studies which have shown early EBV infections among young children in Africa.^17^,^29^ However, EBV prevalence was higher in blood donors from rural areas (9.4%) compared to those from urban areas (5.2%) but the difference observed is not statistically significant.

The high prevalence of EBV in blood donors questions the transmission of this virus through blood transfusion not only in immunodepressed patients, but also in patients who are carriers of *Plasmodium falciparum*. In immunocompromised subjects, the possible reactivation of the virus causes a higher risk of Burkitt lymphoma.^30^ In subjects carriers of *Plasmodium falciparum* there is a loss of viral control when the anti-EBV immunity is altered.^31^

This study found a high prevalence of infection with blood-borne pathogen in the blood donors’ population. It is necessary to conduct an epidemiologic investigation to evaluate the residual risk in recipients. Other, the multiplex PCR could be an alternative to reduce the high cost of using molecular techniques inpoor countries such as Burkina Faso.

## Conclusion

This study reports for the first time HGV and EBV prevalence in Burkina Faso among blood donors. Further studies will help to define their impact on infected humans.

## Figures and Tables

**Table 1:** Sociodemographic characteristics of blood donors.

Characteristics	Number	Percentage
**Gender**		
Male	464	84.2
Female	87	15.8

**Age group (years)**		
<20	97	17.6
20–29	341	61.9
30–39	88	16.0
≥40	25	4.5

**Place of blood donation**		
Urban areas	425	77.1
Rural areas	126	22.9

**Blood groups**		
O	242	43.9
A	133	24.1
B	143	26.0
AB	33	6.0

**Rhesus (Rh) type**		
Positive	511	92.7
negative	40	7.3

**Table 2:** Prevalence of HGV and co-infections among first time blood donors.

Infections	Number of positive	percentage
HGV	41	7.4
HBV	71	12.9
HCV	18	3.3
HIV	12	2.2
Syphilis	15	2.7

**Co-infections**		
HBV-HGV	31	5.6
HGV-HCV	5	0.9
HGV-syphilis	2	0.4

**Table 3:** Sociodemographic characteristics by HGV and EBV among blood donors.

Characteristics	Total	HGV Positive	OR (95% CI)	P-values	EBV Positive	OR (95% CI)	P-values
Gender							
Male	464	35 (7.5)	0.9 (0.3–2.4)	0.99	28(6.0)	2.7(0.6–17.0)	0.202
Female	87	6 (6.9)	1		2 (2.3)	1	

Age group (years)							
<20	97	6 (6.2)	1		19(19.6)	11.6(4.4–31.7)	<0.001
20–29	341	27(7.9)	1.3 (0.5–3.6)	0.73	7 (2.1)	1	
30–39	88	7 (9.0)	1.5 (0.5–5.1)	0.66	3 (3.4)	1.6(0.3–7.4)	0.435
≥40	25	0 (0.0)	-	-	1 (4.0)	NA	-

**HCV status**							
Negative	533	36 (6.8)	1	-	-	-	-
Reactive	18	5 (27.8)	5.3 (1.6–17.2)	0.004	-	-	-

**HBV status**							
AgHBs negative	480	22 (4.6)	1	-			
AgHBs positive	71	19 (26.7)	7.6 (3.7–15.8)	<0.001	-	-	-

**Place of blood donation**							
Urban areas	425	33 (7.8)	1.2 (0.5–3.0)	0.74	20 (4.7)	1	
Rural areas	126	8 (6.4)	1	-	10(7.9)	1.8(0.7–4.1)	0.238
Total	551				30(5.4)		
